# Associations between the Severity of the Post-Acute COVID-19 Syndrome and Echocardiographic Abnormalities in Previously Healthy Outpatients Following Infection with SARS-CoV-2

**DOI:** 10.3390/biology10060469

**Published:** 2021-05-26

**Authors:** Cristina Tudoran, Mariana Tudoran, Gheorghe Nicusor Pop, Catalina Giurgi-Oncu, Talida Georgiana Cut, Voichita Elena Lazureanu, Cristian Oancea, Florina Parv, Tudor Ciocarlie, Felix Bende

**Affiliations:** 1 Department VII, Internal Medicine II, Discipline of Cardiology, University of Medicine and Pharmacy “Victor Babes” Timisoara, E. Murgu Square, Nr. 2, 300041 Timisoara, Romania; tudoran.cristina@umft.ro (C.T.); parv.florina@umft.ro (F.P.); ciocarlie.tudor@umft.ro (T.C.); 2 Center of Molecular Research in Nephrology and Vascular Disease, Faculty of Medicine, University of Medicine and Pharmacy “Victor Babes” Timisoara, E. Murgu Square, Nr. 2, 300041 Timisoara, Romania; 3 County Emergency Hospital Timisoara, 300041 Timisoara, Romania; catalina.giurgi@umft.ro (C.G.-O.); bende.felix@umft.ro (F.B.); 4 Department VI, Cardiology, University of Medicine and Pharmacy “Victor Babes” Timisoara, E. Murgu Square, Nr. 2, 300041 Timisoara, Romania; pop.nicusor@umft.ro; 5 Department VIII, Neuroscience, Discipline of Psychiatry, University of Medicine and Pharmacy “Victor Babes” Timisoara, E. Murgu Square, Nr. 2, 300041 Timisoara, Romania; 6 Department XIII, Discipline of Infectious Diseases, University of Medicine and Pharmacy “Victor Babes” Timisoara, E. Murgu Square, Nr. 2, 300041 Timisoara, Romania; talida.cut@umft.ro (T.G.C.); vlazureanu@gmail.com (V.E.L.); oancea@umft.ro (C.O.); 7 Department of Gastroenterology and Hepatology, University of Medicine and Pharmacy “Victor Babes” Timisoara, 300041 Timisoara, Romania; 8 Center of Advanced Research in Gastroenterology and Hepatology, Faculty of Medicine, University of Medicine and Pharmacy “Victor Babes” Timisoara, 300041 Timisoara, Romania

**Keywords:** COVID-19, post-acute COVID-19 syndrome, cardiovascular abnormalities, transthoracic echocardiography, quality of life

## Abstract

**Simple Summary:**

With the COVID-19 pandemic lasting over a year and affecting all continents, a new problem has arisen—that of convalescents, who continue to have various symptoms at more than 4 and up to 12 weeks after the acute disease, the so called post-acute COVID-19 syndrome. In this article, we tried to determine if previously healthy adults with post-acute COVID-19 syndrome also have cardiac complications related to the number of persisting symptoms, the quality of life scores and the initial pulmonary injury. Using transthoracic echocardiography, we found cardiac abnormalities (pulmonary hypertension, systolic and diastolic dysfunction, pericarditis) in about a quarter of the 150 participants in our study. Their gravity was significantly correlated with the severity of COVID-19, the number of weeks passed since the acute illness, the number of persisting symptoms, and the quality of life. Post-acute COVID-19 is a recently proposed term which aims to characterize the various symptoms persisting after an acute SARS-CoV-2 infection, their severity being explained partially by residual multi-system alterations, with an important impact on the functional status and quality of life of the affected individuals.

**Abstract:**

The COVID-19 pandemic affected over 130 million individuals during more than one year. Due to the overload of health-care services, a great number of people were treated as outpatients, many of them subsequently developing post-acute COVID-19 syndrome. Our study was conducted on 150 subjects without a history of cardiovascular diseases, treated as outpatients for a mild/moderate form of COVID-19 4 to 12 weeks prior to study inclusion, and who were diagnosed with post-acute COVID-19 and attended a cardiology evaluation with transthoracic echocardiography (TTE) for persisting symptoms. We detected various cardiac abnormalities in 38 subjects (25.33%), including pulmonary hypertension (9.33%), impaired left ventricular performance (8.66%), diastolic dysfunction (14%) and/or evidence of pericarditis (10%). We highlighted statistically significant correlations between the intensity of symptoms and quality of life scores with the severity of initial pulmonary injury, the number of weeks since COVID-19 and with TTE parameters characterizing the systolic and diastolic performance and pulmonary hypertension (*p* < 0.001). (Post-acute COVID-19 is a complex syndrome characterized by various symptoms, the intensity of which seem to be related to the severity and the time elapsed since the acute infection, and with persisting cardiac abnormalities.

## 1. Introduction

As the SARS-CoV-2 infection spreads unabated worldwide, inducing second and even third outbreaks of COVID-19, it has proved to be the most significant pandemic of the last centuries—over 130 million individuals have been affected and the global health systems are overwhelmed [1]. In line with the increasing number of patients, more and more of them continue to describe symptoms like fatigue, palpitations, decreased exercise capacity, shortness of breath, chest pain, neurocognitive difficulties, muscle pains and weakness, depression, anxiety and other mental health conditions, which appear to persist weeks after the acute phase of the viral infection [2,3]. In their study, Greenhalgh et al. defined this syndrome as post-acute COVID-19 if symptoms persist between 3 and 12 weeks after the onset of the disease, and as long COVID-19 if symptoms last over 3 months [3,4]. Its reported incidence varies largely between different studies, ranging from 10% to 50% of cases [3,5]. In contrast to post-acute syndromes developed following other severe infections, the particularity of the post-acute COVID-19 syndrome is that it has been described even in patients who suffered mild/moderate forms of the infection, not requiring admission to the intensive care unit [2,3]. The mechanisms responsible for the post-acute COVID-19 syndrome are not entirely understood. Some authors have suggested a role of persistent viremia due to weak or absent antibody response, inflammation and immune reactions, deconditioning, post-traumatic stress or even relapse and/or reinfection with SARS-CoV-2 [3]. These factors could favor the occurrence of myocardial injury and inflammation, which could subsequently induce left ventricular dysfunction [3].

The management of post-acute COVID-19 raises various challenges, since there are no clear guidelines available. To our knowledge, there are currently only a few studies focusing on the comprehensive evaluation of patients with post-acute COVID-19, especially of the associated cardiovascular (CV) involvements assessed by transthoracic echocardiography (TTE), in order to establish to what extent the remaining alterations could be responsible for the persistence of symptoms [6,7,8]. Additionally, there were some attempts to elaborate scales for the classification of the functional status of patients with post-acute COVID-19 [9,10] but their subsequent management remains a matter of debate.

The aim of this study was to establish the potential associations between the number of symptoms characterizing the post-acute COVID-19 syndrome and the quality of life (QoL) scores at 4 to 12 weeks from the acute phase with cardiac dysfunction, as diagnosed in these patients with the use of TTE. Another aim was to determine if there is any relationship between these remaining cardiac abnormalities and the severity of COVID-19, expressed by the magnitude of the initial pulmonary injury and the inflammatory response.

## 2. Materials and Methods

### 2.1. Study Population

Study population: Our study included 150 patients who suffered from a SARS-CoV-2 infection during the second outbreak of COVID-19 in the West of Romania (between 1 August 2020 and 31 January 2021), when hospitalization was not mandatory for all infected individuals. Patients were selected randomly from all of the subjects with a SARS-CoV-2 infection, who attended the outpatient cardiology or internal medicine services of our hospitals for certain symptoms (e.g., persistent fatigue, shortness of breath, chest discomfort/pain, palpitations, reduced effort capacity). Our study only includes patients who suffered from a mild/moderate form of COVID-19 4 to 12 weeks prior to clinic attendance, and who underwent a COVID-19 evaluation, including thoracic computer tomography (TCT) and blood tests, on an outpatient basis. We selected 150 suitable outpatients who signed individual informed consent forms prior to the collection of any data. At the inclusion in this study, their baseline clinical characteristics, TCT and laboratory data, as well as their pre-existing TTE results were registered from their medical records. Patients were asked to complete a QoL questionnaire, and their functional status was assessed according to the Post-COVID-19 Functional Status (PCFS) scale. Subsequently, all subjects underwent a clinical exam, and electrocardiogram (ECG) and TTE were also performed.

Inclusion criteria: Patients aged over 18 years, with a SARS-CoV-2 infection confirmed by a positive result in real-time reverse transcriptase–polymerase chain reaction (RT-PCR) assay of nasal and pharyngeal swabs, with a COVID-19 evaluation consisting of TCT assessment and laboratory tests, who self-monitored their oxygen saturation by pulse-oximetry at home, and without a pre-existing history of significant CV diseases.

Exclusion criteria: Patients aged over 55 years (with a higher likelihood of suffering from chronic CV pathology or having age-related abnormalities on the TTE), subjects hospitalized for a COVID-19 pulmonary infection, those with severe/critical forms and severe respiratory insufficiency, those without a TCT assessment of the severity of their lung injury, patients with a history of CV diseases or diagnosed during the study with significant cardiac pathology and those without a pre-existing TTE assessment.

The Local Scientific Research Ethics Committee of our hospital approved the design and methodology of our study (No. 206/7.09.2020).

### 2.2. Methods

Initial COVID-19 evaluation: All subjects underwent a first COVID-19 evaluation on an outpatient basis and were classified with mild/moderate forms of the SARS-CoV-2 infection [1,4]. Based on the TCT results, all patient with a pulmonary lesion were classified with mild (<30% pulmonary injury) or moderate (30–60% lesions) forms [11]. This evaluation included laboratory determinations, namely, complete blood cell count (CBC), C-reactive protein (CRP), fibrinogen, aspartate aminotransferase (AST) alanine aminotransferase (ALT) and ferritin.

The EQ-5D-5L QoL questionnaire is a survey designed by the EuroQoL group, covering 5 domains of health (mobility, self-care, ability to perform usual activities, pain/discomfort and anxiety/depression), with 5 levels of severity (no problems, slight problems, moderate problems, severe problems and extreme problems). The participants in our study were asked to rate their current health status according to the EQ visual analogue scale (VAS) [12,13].

The PCFS scale is a methodology proposed to evaluate the recovery after COVID-19, which we applied to the entire patient group in order to identify the severity of their functional limitations, where 0 means “no limitations and symptoms”, 1 signifies “negligible limitations of usual activities with persistent symptoms”, 2 indicates “slight limitations with significant symptoms”, 3 indicates “moderate limitations and not able to perform all usual activities due to symptoms, but still able to take care of themselves without assistance” and 4 signifies “severe limitation due to symptoms and requiring assistance to take care of themselves” [10].

The cardiologic examination included a detailed medical history, a clinical evaluation, ECG and a comprehensive TTE. All measurements were performed according to guidelines’ recommendations [8,14,15]. After a regular examination of the cardiac morphology and function, we determined the left ventricular mass index (LVMI) to evidence left ventricular hypertrophy (LVH), defined by LVMI > 115 g/m^2^ for males and 95 g/m^2^ for females; the left atrial volume index (LAVI), values over 34 mL being considered pathological and the presence of pericardial effusion and of thickened pericardium of over 4 mm and between 2 and 4 mm. Subsequently, the following evaluations were performed:

The evaluation of left ventricular (LV) function (from an apical 4-chamber view) included the assessment of the LV ejection fraction (LVEF) by the modified Simpson rule, with values under 50% being considered abnormal; the measurement of the lateral mitral annular plane systolic excursion (MAPSE), with values < 10 mm being considered pathological; and the quantification of the LV global longitudinal strain (LV-GLS), which was achieved from apical 2, 3 and 4-chamber views. The region of interest was automatically generated, and after tracing the LV endocardial border, manual corrections were performed to fit the thickness of the LV myocardial wall [16,17,18] (pathologic values were defined as LV-GLS > −18%).

The assessment of diastolic dysfunction (DD) was conducted in pulsed Doppler, in apical 4-chamber view, by registering the mitral inflow at the level of the mitral valve annulus, with the peak early diastolic velocity (E), the late diastolic velocity (A) and the assessment of the E/A ratio. Successively, tissue Doppler imaging (TDI) was used to record the early diastolic velocity (e′) and the late diastolic velocity at the level of the septal and lateral mitral annulus, and an average E/e′ ratio was calculated. A type I DD was defined by an E/A ratio ≤ 0.8 and E < 50 cm/s, while a type III DD was confirmed by an E/A ratio of over 2. An E/A ratio ≤ 0.8 with E > 50 cm/s, or an E/A between 0.8 and 2, indicated a type II DD, which was certified if at least two of the following criteria were present: an average E/e′ > 14, LAVI > 34 mL/m^2^ and/or TRV > 2.8 m/s. In cases when only one of these three criteria was fulfilled, a type I DD was diagnosed [15].

Right ventricular (RV) performance was evaluated from a 4-chamber view, by visualizing the entire RV, and included the assessment of the tricuspid annular plane systolic excursion (TAPSE) at the level of the lateral tricuspid valve annulus in M-Mode, with values < 17 mm defining RV dysfunction (RVD); the fractional area change (FAC), with values < 35% being considered pathological; and the RV global longitudinal strain (RV-GLS) performed in apical 4-chamber view, with values > −28% certifying RVD [16,17,19]. The estimated systolic pressure in the pulmonary artery (sPAP) was calculated based on the peak tricuspid regurgitation velocity (TRV), recorded by continuous-wave Doppler, taking into account the right atrial pressure, determined by measuring the inferior vena cava diameter and its respiratory variations. In this study, we considered that sPAP values of ≥35 mmHg at rest indicated pulmonary hypertension (PH), with severities ranging from mild (35–44 mmHg) to moderate (45–60 mmHg) to severe (>60 mmHg) [14,16,17].

Statistical analysis: We utilized the Statistical Package for the Social Sciences v.25 (SPSS, Chicago, IL, USA) to perform data analysis. We presented continuous variables as mean and standard deviation (SD) or median and interquartile range (IQR), and categorical variables as frequency and percentage. Because the results of the normality test (Shapiro–Wilk) showed a non-Gaussian distribution, we continued the analysis using nonparametric tests. For comparing the two groups of patients, we employed the Mann–Whitney U test. To evaluate the significance of differences in the proportions of nominal variables we applied the Chi-square test or Fisher’s exact test. We employed Spearman’s correlation test in order to assess the potential connection between the number of symptoms and the QoL scores with other TTE parameters and laboratory results. We built several multivariate regression models in order to assess the individual impacts of several confounding factors on the variance of continuous variables, and the model was validated based on the accuracy of prediction and R squared. In the final regression equations, the predictors were accepted according to a repeated backward-stepwise algorithm (inclusion criteria *p* < 0.05, exclusion criteria *p* > 0.10) so as to obtain the most appropriate theoretical model that fit the collected data. We considered that *p*-values under 0.05 indicated statistically significant differences.

## 3. Results

The study group included 150 patients, 63 men and 87 women, aged between 18 and 55 years, median age 42 (34.75–50) years, who suffered from a mild/moderate form of COVID-19, the diagnosis being confirmed by a positive result of real-time reverse transcriptase–polymerase chain reaction (RT-PCR) assay of nasal and pharyngeal swabs. For various reasons, these patients were not hospitalized during the acute disease, but all of them had a basal assessment. Most of them, 111 (74%), had mild forms not requiring in-hospital stay; however, there were 39 (26%) with moderate forms, who refused hospitalization. Forty-nine patients (32.66%) were overweight and 44 (29.33%) were obese. It should be mentioned that, according to the PCFS scale, 94 patients were classified as grade 1, 44 as grade 2 and 12 as grade 3. Referring to the EQ-5D-5L QoL questionnaire results, none of our patients reported experiencing a limitation of mobility and self-care, but noted limitation in their usual activities, persistence of discomfort, as well as symptoms of anxiety and/or depression of various severities.

**Figure 1:**
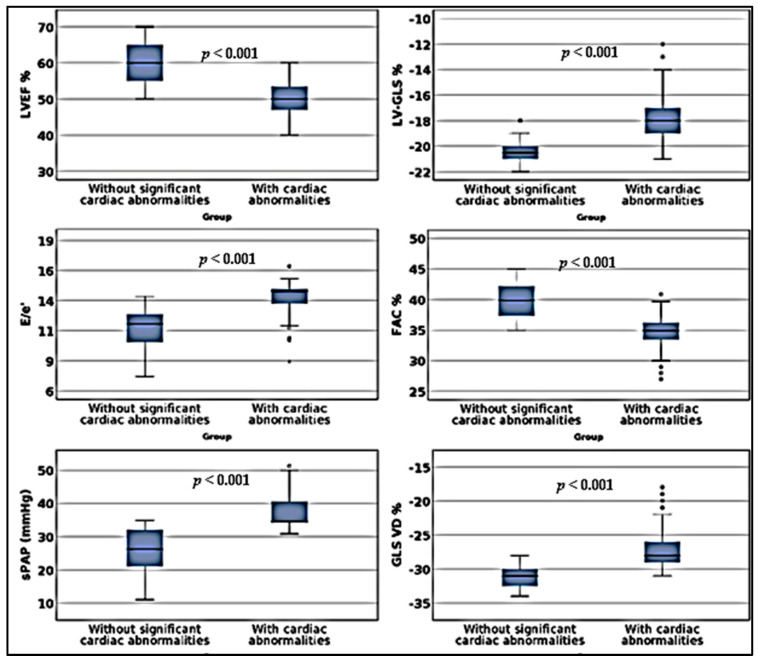
Prevalence of cardiac abnormalities in study groups. LVEF: left ventricular ejection fraction; LV-GLS: left ventricular global longitudinal strain; E/e′: early mitral inflow diastolic velocity E to average e′ velocity (E/e′) in pulsed tissue Doppler; FAC: fractional area change; sPAP: systolic pressure in the pulmonary artery; RV-GLS: right ventricular global longitudinal strain.

Following the clinical examination and a comprehensive TTE assessment, there was no evidence of significant cardiac abnormalities in 112 patients (74.66%), 48 men and 64 women, except for trivial mitral or aortic regurgitation and a mild LVH in one woman. In 38 other subjects, significant CV alterations were diagnosed. The latter patient subgroup was significantly older and had higher BMIs; they had more severe pulmonary injuries, higher values of CRP and were more symptomatic during the acute phase of COVID-19, which might explain why they attended a medical consultation sooner (Table 1, Figure 1). These patients were classified with higher PCFS scores, they reported worse QoL with more severe limitations of activity, higher intensities of anxiety/depressive symptoms, and lower VAS scores.

From this subgroup, 13 patients (8.66%) had LVEF values of under 50% and lower LV-GLS (under −18), while 11 of them also showed reduced MAPSE. Of these patients, 11 had increased values of PAPs and associated RVD. Five patients had E/A ratio over 2, thus a type III DD; another 3 patients had E/A ratio under 0.8, thus a type I DD. Of the remaining 5 patients, only 1 was classified with a type I DD, while the other 4 were classified with a type II DD. In 6 cases, there was evidence of pericardial effusion; a pathologically thickened pericardium (over 4 mm) was detected in another 3 patients.

Following the TTE evaluation, we identified 14 patients (9.33%) with increased PAPs, all of them having an associated RV dysfunction. It is worth mentioning that we also detected alterations of RV performance in a further 4 subjects, 2 of whom had concomitant reduced LVEF and another 2 showed a type II DD.

In a subset of 21 subjects (6 men and 15 women) with LVH, we assessed an increased E/e′ ratio of over 14, certifying the presence of DD; 8 of them had an E/A ratio under 0.8, and the remaining 13 showed values over 0.8 (but under 2). Of these 22, 7 subjects also had increased LAVI, thus a type II DD was diagnosed; the remaining 14 patients were classified with a type I DD. One patient presented with pericardial effusion and 3 others had thickened pericardium of over 4 mm.

When referring to the results of the EQ-5D-5L QoL questionnaire, none of our patients reported reduced mobility or limited self-care, but most of them claimed slight, moderate or severe restrictions of usual activities, persisting discomfort and anxiety/depression.

By analyzing the existence of potential associations between the number of remaining symptoms and the VAS score, we evidenced statistically significant correlations between the severity of pulmonary injuries and levels of CRP during the acute phase of COVID-19 and with the number of weeks elapsed since its diagnosis. We also found significant correlations with TTE parameters such as sPAP defining PH; with FAC, RV-GLS and TAPSE characterizing RVD; with LV-GLS, LVEF and MAPSE suggestive of LV impaired systolic performance; and even with E/e′ in patients with and without substantial cardiac alterations (Table 2).

In the absence of specific guidelines, these patients were treated according to the recommendations specific for the identified dysfunctions: pericarditis was treated with non-steroidal anti-inflammatory agents (Ibuprofen 400 mg twice/day), left ventricular systolic dysfunction with beta-blockers (especially in the presence of associated tachycardia), and/or small doses of angiotensin-converting enzyme inhibitors. In some cases with pulmonary hypertension who raised concerns that pulmonary microthrombosis could be implicated, the anticoagulant therapy was prolonged.

**Figure 2:**
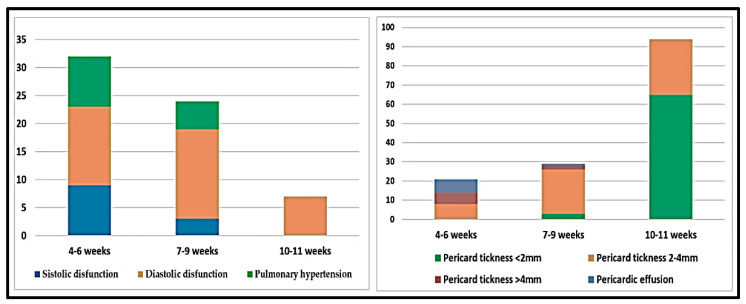
Time distribution of cardiac abnormalities assessed by TTE.

The results of our TTE evaluation varied significantly in line with the number of weeks since the acute disease. Thus, only 30 patients were evaluated in the first 4 to 8 weeks after COVID-19, which led to diagnoses of PH and LVD, associated with DD of type II or III, in 12 patients. We evidenced the presence of a small pericardial effusion (between 3.5 and 5 mm) in 8 subjects, and thickened pericardium of over 4 mm in an additional 7 cases. Seven patients had only type I or II DD. The remaining 120 patients were evaluated after 9 and up to 11 weeks of recovery, resulting in a diagnoses of PH in only 2 subjects, one of them also having a reduced LV systolic performance. DD was evidenced in 14 subjects, all of them having associated LVH and increased BMI. In these patients we frequently found a thickened pericardium of over 2 mm, and between 2 and 4 mm (9.33%). The distribution of these abnormalities is depicted in Figure 2.

To establish which are the most significant predictive factors that could influence the number of remaining symptoms in our patient group with post-acute COVID-19 syndrome, we employed a multivariate linear regression analysis. We used the forward stepwise method, and the best model was selected based on Akaike information criteria (AICs). We determined that the number of weeks elapsed since the onset of the COVID-19 infection, the PCFS score and the RVD characterized by RV-GLS explained 87.6% of the remaining symptoms (R^2^ = 0.876, β = −0.458, S.E. = 0.42, *p* < 0.001).

## 4. Discussion

With the COVID-19 pandemic lasting for over a year, more and more people became infected with SARS-CoV-2, overwhelming health services to such a degree that only severe cases were hospitalized, while the majority had to be treated as outpatients [2,20]. Subsequently, the persistence of symptoms weeks after the acute phase has been observed in survivors of severe forms of this illness, but also in individuals who have suffered from moderate or even mild forms of COVID-19 [21]. At first, these symptoms were considered to be solely related to the sequelae of pulmonary injuries, but since similar manifestations have been described even in the absence of COVID-19 pneumonia, some authors recognized that there must be an entire syndrome responsible for these dysfunctions, with several organs being affected—especially the heart, lungs and brain [2,3].

Largely, it has been considered that CV complications that are frequently associated with COVID-19 could at least partially explain symptoms such as fatigue, shortness of breath, palpitations, chest pain and reduced exercise capacity. The diagnosis of these complications can be established relatively easy by TTE. The reported incidence depends on the studied category of patients and on the pre-existing CV pathology [8]. Szekeley et al. describe an increased prevalence of RVD (observed in 39% of patients), followed by DD (16%), and an altered systolic function (10%) [22]. In our study, we highlighted an increased prevalence of PH (9.33%), frequently associated with RV dysfunction LVD (8.66%), in patients with post-acute COVID-19 syndrome without significant pre-existing CV pathology. However, these alterations were diagnosed mostly in subjects with more severe pulmonary injury, who were examined earlier after the acute phase of COVID-19, and were strongly associated with having more symptoms, higher PCFS scores and a lower QoL. DD was more frequently diagnosed in overweight or obese subjects (14%), the majority of them also showing LVH, and it was evidenced mostly in patients examined between 9 and 11 weeks after the COVID-19 infection, sustaining the hypothesis that it could be related to fibroses following an acute inflammatory process of the myocardium, which is also supported by magnetic resonance imaging (MRI) studies [2,10,13]. Another interesting finding was the presence of pericardial effusion evidenced early (at 4 to 8 weeks after the acute phase), while only a thickened pericardium was detected in the subjects who were evaluated later, suggesting an immunologically mediated late pericarditis [23,24,25].

Some authors state that the majority of patients with post-acute COVID-19, especially those with mild/moderate forms, recover completely after a period of time, usually after 4–6 weeks; however, others report the persistence of symptoms even after more than 12 weeks, advancing terms such as long COVID [3,20]. In our results, we noticed that the severity of cardiac alterations diminished in parallel with the time elapsed since the diagnosis of COVID-19. The current evaluation (as in the case of PH, RVD or impaired LV systolic function) revealed that other acute aspects such as pericardial effusion were diagnosed only in the first 6 weeks following the acute illness, while other abnormalities such as DD and thickened pericardium were detected in subjects who were examined later.

However, our study also evidenced significant associations between the number of remaining symptoms and the QoL scores with the severity of the initial pulmonary lesions, with the number of weeks elapsed since the onset of symptoms, with the functional class and with the severity of cardiac alterations [26].

Some authors highlighted the important role of cardio-pulmonary rehabilitation programmes [27], with exercise training being a powerful tool in physiotherapy, having the capacity to induce significant improvements in the respiratory and CV systems, facilitating the functional recovery of the respiratory musculature and of the cardiac performance, as well as the reduction of the endothelial dysfunction and of thromboembolic complications [28]. Another aspect is the impact of cardio-pulmonary rehabilitation on the functional capacity, quality of life and prognosis [29].

It is therefore important that healthcare services establish guidelines for the comprehensive multidisciplinary evaluation and follow-up of COVID-19 patients to provide a timely diagnosis and therapy of residual complications, as well as support for the gradual recovery of patients suffering from post-acute COVID-19.

## 5. Conclusions

Post-acute COVID-19 is a complex syndrome including a large spectrum of persistent symptoms, frequently explained by residual cardiac abnormalities, but also by mental health disturbances whose gravity seems to be related to the initial severity of COVID-19 and with the time elapsed since the acute infection, with a long-term impact on the functional status and the quality of life of the numerous individuals affected by this disease.

## Figures and Tables

**Table 1:** Demographics, clinical and laboratory data in the patient group.

Characteristics of Patients		Without Significant Cardiac Abnormalities 112 (74.66%)	With Cardiac Abnormalities 38 (25.33%)	*p*
Age (years)		39.52 (31–46.75)	48.86 (46.75–53.25)	<0.001 ^a^
Gender	male 63	48	15	0.715 ^b^
	female 87	64	23	
BMI kg/m^2^		26.63 (23.53–29.41)	30.48 (28.34–32.77)	<0.001 ^a^
Severity form:	mild	110	1	<0.001 ^c^
	moderate	2	37	
O_2_ saturation during COVID-19%		97.55 (97–99)	94.73 (94–96)	<0.001 ^a^
TCT assessed injury (%)		0 (0–0)	30.5 (30–35)	<0.001 ^a^
Initial CRP (mg/dL)		22.1 (18.54–29)	36.58 (31–41.75)	<0.001 ^a^
Heart rate (b/min)		73 (70–80)	75 (75–80)	0.126 ^a^
No. of persisting symptoms		2.59 (2–3)	4.47 (3–6)	<0.001 ^a^
Weeks since COVID-19		10.08 (10–11)	7.44 (5.75–9)	<0.001 ^a^
PCFS score		1 (1–1)	2 (2–3)	<0.001 ^a^
Quality of life—VAS score		76.28 (70–82)	62 (55–69)	<0.001 ^a^
Echocardiographic parameters				
Pericardial abnormalities				
Thickness		1.5 (1.42–2.3)	3.57 (2–5.1)	<0.001 ^a^
Exudate		-	8	0.004 ^c^
LVMI		86.84 (75.53–96.2)	105 (97.27–114.73)	<0.001 ^a^
LAVI		17.42 (13.45–20.16)	30.19 (27.91–34.56)	<0.001 ^a^
E/A		1.15 (0.96–1.33)	1.19 (0.75–1.32)	0.216 ^a^
E/e′		11.15 (10–12.36)	13.63 (13.21–14.49)	<0.001 ^a^
TRV		2.26 (2–2.6)	2.84 (2.7–2.98)	<0.001 ^a^
sPAP		26.11 (21–32)	37.5 (34.16–40.64)	<0.001 ^a^
TAPSE		23.35 (21.25–25)	18.34 (15.75–20)	<0.001 ^a^
FAC		39.68 (37.34–42.21)	34.38 (33.17–36.23)	<0.001 ^a^
RV-GLS		−30.9 (−32.75–−30)	−17.741 (−19–−17)	<0.001 ^a^
MAPSE		16.23 (15–18)	12.33 (8.75–15.62)	<0.001 ^a^
LVEF		60 (55–65)	50.48 (47–53.62)	<0.001 ^a^
LV-GLS		−20.43 (−21–−20)	−26.55 (−29–−25.75)	<0.001 ^a^

Legend: BMI: body mass index; TCT: thoracic computed tomography; CRP: C reactive protein; PCFS: Post COVID-19 Functional Status; VAS: visual analogue scale; LVMI: left ventricular mass index; LAVI: left atrial volume index; E/A: peak mitral inflow early (E) to late (A) diastolic velocities in pulsed Doppler; E/e′: early mitral inflow diastolic velocity E to average e′ velocity (E/e′) in pulsed tissue Doppler; TRV: peak tricuspid regurgitation velocity; sPAP: systolic pressure in the pulmonary artery; TAPSE: tricuspid annular plane systolic excursion; FAC: fractional area change; RV-GLS: right ventricular global longitudinal strain; MAPSE: mitral annular plane systolic excursion; LVEF: left ventricular ejection fraction; LV-GLS: left ventricular global longitudinal strain; ^a^ Mann–Whitney U test; ^b^ Chi-square test; ^c^ Fisher’s exact test.

**Table 2:** Correlations between number of symptoms and EQ-5D-5L QoL scores.

Analyzed Parameters	No. of Remaining Symptoms			Quality of Life		
	r	95%CI	*p*	r	95%CI	*p*
Initial TCT injury	0.664	0.548; 0.751	<0.001	−0.670	−0.751; −0.567	<0.001
Initial CRP	0.709	0.609; 0.788	<0.001	−0.761	−0.832; −0.667	<0.001
PCFS score	0.775	0.701; 0.837	<0.001	−0.758	−0.817; −0.681	<0.001
Weeks since COVID-19	−0.856	−0.898; −0.796	<0.001	0.847	0.783; 0.896	<0.001
LVEF	−0.553	−0.671; −0.433	<0.001	0.687	0.587; 0.77	<0.001
LV-GLS	0.606	0.49; 0.707	<0.001	−0.687	−0.77; −0.592	<0.001
MAPSE	−0.433	−0.574; −0.285	<0.001	0.537	0.407; 0.654	<0.001
FAC	−0.78	−0.838; −0.707	<0.001	0.831	0.758; 0.883	<0.001
RV-GLS	0.816	0.53; 0.865	<0.001	−0.895	−0.931; −0.844	<0.001
TAPSE	−0.671	−0.775; −0.554	<0.001	0.725	0.626; 0.805	<0.001
sPAPs	0.791	0.726; −0.837	<0.001	−0.843	−0.885; −0.777	<0.001
E/e′	0.470	0.326; 0.587	<0.001	−0.562	−0.668; −0.420	<0.001

Legend: TCT: thoracic computed tomography; CRP: C reactive protein; PCFS: Post COVID-19 Functional Status; LVEF: left ventricular ejection fraction; LV-GLS: left ventricular global longitudinal strain; MAPSE: mitral annular plane systolic excursion; FAC: fractional area change; RV-GLS: right ventricular global longitudinal strain; TAPSE: tricuspid annular plane systolic excursion; sPAP: systolic pressure in the pulmonary artery; E/e′: early mitral inflow diastolic velocity E to average e′ velocity (E/e′) in pulsed tissue Doppler; Spearman’s correlation.

## Data Availability

Our blinded patient data are available on http://dx.doi.org/10.17632/dnv6fb9x7y.1 (accessed on 22 April 2021).
